# Antioxidant potential of a soft cheese (paneer) supplemented with the extracts of date (*Phoenix dactylifera* L.) cultivars and its whey

**DOI:** 10.5713/ajas.18.0750

**Published:** 2019-02-14

**Authors:** Tahir Mahmood Qureshi, Aniqa Amjad, Muhammad Nadeem, Mian Anjum Murtaza, Masooma Munir

**Affiliations:** 1 Department of Food Sciences, Cholistan University of Veterinary & Animal Sciences, Bahawalpur, 63100, Pakistan; 2 Institute of Food Science and Nutrition, University of Sargodha, Sargodha, 40100, Pakistan; 3 Food Science Research Institute, National Agricultural Research Centre, Islamabad, 44000, Pakistan

**Keywords:** Date Cultivars Extracts, Paneer, Physico-chemical Characteristics, Microbiological Quality, Antioxidant Potential

## Abstract

**Objective:**

The present study was conducted to evaluate the antioxidant potential of paneer, a soft cheese supplemented with various water soluble date extracts during storage. Further, the whey obtained from all the paneer samples was also investigated for its antioxidant potential.

**Methods:**

The date cultivars were evaluated for their physico-chemical characteristics and date extracts were assessed for their antioxidant potential. Physico-chemical evaluation, microbiological quality and further antioxidant potential of the prepared paneer were carried out during storage period (0 to 8 days, 5°C).

**Results:**

All the date extracts were found to have considerable antioxidant activity due to presence of total phenolics and flavonoids. Owing to the presence of phenolics and flavoinds in date extracts, supplemented paneer showed higher trolox equivalent antioxidant capacity, reducing power and 2, 2-diphenyl-1-picrylhydrazyl (DPPH) radical scavenging activity than control paneer. Paneer supplemented with Rabi extracts had the highest total phenolics (190.7 μg gallic acid equivalent/g paneer), DPPH radical scavenging activity (928.1 μmol equivalent of Trolx/g paneer) and trolox equivalent antioxidant capacity (9.2 μmol equivalent of Trolx/g paneer). The whey obtained from control paneer showed lower values of total phenolics, total flavonoids, DPPH, trolox equivalent antioxidant capacity and reducing power as compared to the values of whey obtained from paneer supplemented with date extracts.

**Conclusion:**

Paneer supplemented with date extracts and its whey may offer potent antioxidant activity.

## INTRODUCTION

Paneer is a primitive soft cheese variety which is produced by heat treatment of milk followed by acid coagulation using some suitable acid viz. lactic acid, citric acid, tartaric acid, sour whey, alum. It is used in culinary dishes/snacks and sweets like rasgolla, rasmalai, sandesh etc. It is considered as an important nutritious and wholesome indigenous dairy product, which occupies a prominent place among other traditional milk products and carries market potential [1]. It is considered a rich source of high quality proteins, fat, minerals and vitamins. Different studies concerning antioxidant activity of different cheeses such as cottage cheese [2], Parmigiano-Reggiano cheese [3], traditional Sheep cheese (Bieno Sirenje) [4] and Indian paneer supplemented with peel extracts from pomegranate, lemon and orange [5] have been conducted by many researchers.

The date palm (*Phoenix dactylifera* L., family Arecaceae) fruit is commonly consumed in Middle East and around the world due to its nutritional, economic and medicinal properties [6]. Antioxidant activity and phenolic contents of some date fruit cultivars grown in Saudi Arabia, USA and Oman have already been investigated [7–9].

To most of the people in our culture, paneer is considered a tasteless dairy product. So the addition of date extract into the milk intended for paneer making would be a better option to enhance the bioactivity and palatability. Different date cultivars are available in the local market of Pakistan but the most commonly consumed cultivars selected in the present study were Aseel, Rabi, Irani, and Muzafati. To date, no comprehensive study was conducted regarding physico-chemical characteristics of selected indigenous date cultivars and antioxidant activity of their water soluble date extracts (30°Brix). It was decided to use water soluble date extracts as water soluble (hydrophilic) phenolics (mainly cinnamic acids) and flavonoids (flavones, flavonols, and flavanones) with appreciable free radical scavenging activity and antioxidant activity have been previously reported [10].

Moreover, no literature was found concerning antioxidant potential of paneer supplemented with date extracts. Therefore, in view of the above aspects, the present study was designed to study the physico-chemical characteristics of date cultivars indigenous to Pakistan in a comprehensive manner and further, to evaluate the antioxidant potential of paneer supplemented with water soluble date extracts during storage as well as whey collected from those paneer. Being rich source of major and minor bioactive components [11,12], buffalo milk was used for paneer production.

## MATERIALS AND METHODS

### Chemicals

2, 2-Diphenyl-1-picrylhydrazyl (DPPH), Catechin, Folin-Ciocalteu reagent and Trolox were purchased from Sigma-Aldrich Chemical Co. (St. Louis, MO, USA). Sodium phosphate, aluminium trichloride (AlCl_3_) and sodium carbonate were purchased from Daejung Co. Ltd., Korea. Calcium carbonate was purchased from BDH Laboratory Supplies, England. All other chemicals were of analytical grade.

### Milk and date fruit samples

Buffalo milk was collected from a local farm at Sargodha city and ripened date fruit cultivars i.e. Aseel, Rabi, Irani, and Muzafati were brought from the local market to the Institute of Food Science and Nutrition, University of Sargodha. The raw milk was stored at 4°C for 3 hours before carrying out physico-chemical analysis and production of paneer.

### Physico-chemical analysisof date cultivars

Fruit weight (g) was determined using a digital calibrated analytical balance (Electronic scale JJ224BC; Mettler Toledo, Columbus, OH, USA).Moisture, ash, crude fat, crude fiber, and total protein were determined by the standard methods of the Association of Official Analytical Chemists (AOAC) [13]. The carbohydrate content was determined by subtracting the moisture, crude fiber, fat, total protein and ash content from the total mass (i.e. 100). The estimated energy values were calculated by summing the multiplied values for fat, total protein and carbohydrate by their respective factors (9, 4, and 4). Before evaluating pH and acidity, the fruit flesh of each date cultivar (10 fruits) was mashed with the help of a pestle mortar to form a paste. The pH of each date paste was determined by digital pH meter [13] and the total titratable acidity was determined by titrating 10 mL aliquot of sample (1:10 dilution) with standardized 0.1 N NaOH to an endpoint of pH 8.1. Phenolphthalein was used as an indicator and the results were expressed as grams of citric acid/100 mL of sample [13]. Following formula was used to calculate acidity of samples:

$$\text{Acidity }\%=\frac{64\;(\text{MW})\times 0.1\text{N }(\text{NaOH})\times \text{Titer value }(\text{mL})}{1000\times 10\;(\text{aliquot taken})}\times 100$$

### Preparation of date extracts

All the date cultivars (Aseel, Rabi, Irani, and Muzafti) were washed thoroughly with tap water and then pitted and cut into small pieces. They were steamed (90°C to 95°C for 10 min) to prevent growth of bacteria, mould and for inactivation of any enzyme and to make the flesh soft. Mincing of the cut pieces was done by mincing machine, which converted them into paste. Then the paste was heated after addition of water (1:1) at 60°C to 70°C for 2 to 3 hours. The heated paste was then cooled and blended by using a blender. The slurry was filtered through a muslin cloth to separate the date extract from the fruit flesh. The brix of all extracts was maintained at around 30°Brix by the removal of more moisture at 60°C to 70°C through water bath. The obtained extracts were saved in bottles at refrigeration (4°C) and used the next day in making paneer. The date extracts were also centrifuged at 14,000 g for 10 min in eppendorf centrifuge (Hermle Labortechnik GmbH Siemensstr-25 D-78564 Wehingen, Germany) at 4°C. The clear supernatant was taken for carrying out antioxidant potential.

### Physico-chemical analysis of milk and date extracts

Fresh raw milk was standardized for fat and its solids not fat (SNF) were calculated using a lactometer. The pH of date extracts and milk was monitored using a pH meter after calibrating with buffer of pH 4.0 and pH 7.0 [14]. The fat in milk was determined by the Gerber-van Gulik method using a butyrometer [14]. The total soluble solid of date extracts was determined by using a calibrated Atago Analog Refractometer [13]. Vitamin C, total sugars, reducing sugars and non-reducing sugars, of date extracts were determined according to AOAC [13].

### Sample preparation

The date extracts (~30°Brix) from all the cultivars and milk were blended at the ratio of 20:80 for the production of paneer. In our preliminary trials, date extracts and milk were blended in different ratios but the above mentioned ratio was selected on the basis of better sensory evaluation of final product. Control (paneer) treatment (only milk without addition of date extract) was designated as P_0_. The treatment denoted as P_1_ was the paneer prepared from the blend of Aseel extract and milk whereas P_2_ was the treatment (paneer) prepared from the blend of Muzafati extract and milk. Paneer manufactured from Irani extract and milk was denoted by P_3_ whereas P_4_ depicted the paneer obtained from the blend of Rabi extract and milk.

### Production of paneer supplemented with date extract

Paneer was produced according to the method as described by Khan et al [15] with some modifications. For all treatments, the blend (8 L) of each date extract and milk was heated up to 82°C for 5 min and cooled to 70°C. At this stage, coagulant (2% citric acid) was added with continuous but gentle stirring. The obtained coagulum was left undisturbed for approximately 5 to 7min and the temperature of the contents was allowed to drop until 60°C. The whey was drained using a muslin cloth. The coagulum was then filled in a stainless steel mold with holes on all sides to facilitate quick and efficient removal of the whey. The mold was lined with muslin cloth from inside and the whole paneer mass was then pressed by a hydraulic press (1 bar pressure) for 15 min.

### Sampling of paneer

Each paneer was then carefully cut into four identical pieces. One piece was stored at 5°C for 4 days and another for 8 days. One piece from each treatment was frozen immediately (0 day) until analyzed. In a preliminary trial, it was observed that the paneer samples stored for more than 10 days lost their freshness and started to deteriorate. In short, paneer samples stored for more than 12 days were not acceptable sensorially and therefore, they were not included in the study. All the pieces were wrapped first in a polyethylene film and then covered by an aluminum foil. All the treatments (P_0_, P_1_, P_2_, P_3_, P_4_) were prepared in the same manner three times. In this way, three replicates were obtained for each treatment at each storage period.

### Preparation of water soluble extracts of paneer

The procedure described by Gupta et al [16] with some modifications was adapted for the preparation of water soluble extracts (WSEs) of all paneer samples. Crushed paneer sample (20 g) was mixed with 40 mL of distilled water. To make homogenous mixture, the sample was sonicated (20 kHz frequency, 70% amplitude level (525Wpower), and pulse duration 5 s on and 5 s off, 2 min at 20°C) using an ultrasonic processor (UP400S, Hielscher Ultrasonics GmbH Hielscher USA, Inc., Mount Holly, NJ, USA) with a probe inserted up to 2 inch inside the sample. The mixture was centrifuged (Hermle Labortechnik GmbH Siemensstr-25 D-78564, Germany) at 14,000 g for 15 min at 4°C after adjusting pH 4.6 by using 1 N HCl. The fat layers were removed and the supernatants were filtered through Whatman No.1 filter paper (Merck KGaA, Darmstadt, Germany). The aliqouts of the WSEs were immediately frozen at −10°C. Finally, before carrying out antioxidant potential assays, the WSEs were filtered through 0.45 μm pore size filter (Microlab Scientific Co., Ltd, Hongkong, China).

### Proximal analysis of prepared paneer

For the analysis of paneer, it was cut to get identical sampled pieces according to the International Dairy Federation (IDF) standard 50C [17]. The pH of grated paneer was monitored using a pH meter. The electrode of pH meter was placed in the grated paneer with a few drops of water [14]. Acidity of paneer was determined by AOAC [13]. Moisture contents of paneer was determined according to IDF standard 4/ISO 5534 [18] through gravimetric method. The fat content was determined by the Gerber-van Gulik method using a butyrometer [14]. Total nitrogen (%) was estimated according to the IDF standard 20B [19]. Further, protein contents (%) were calculated by multiplying total nitrogen (%) with 6.38. The ash content was estimated by AOAC [13]. Fat as a percentage of dry matter was also calculated from the values of fat and dry matter.

### Chemical characteristics of whey

The pH of whey obtained from control paneer and paneer supplemented with date extracts was monitored [14]. Vitamin C, acidity, total sugars, reducing sugars and non-reducing sugars of all the whey were determined according to AOAC [13].

### Microbiological analysis of prepared paneer

Total plate count and counts for yeast and mould (Y & M) were carried out by following method as described by Broadbent et al [20] with some modifications. Before doing microbiological analysis, all the paneer samples were crushed in pestle and mortar. Mashed sample (10 g) was homogenized with a domestic blender for 5 min after adding 90 mL of sterilized sodium citrate (2%, pH 7.5, warmed to 45°C) water. Serial dilutions from the above suspension were prepared up to10^−4^. One mL of serially diluted samples was plated on plate count agar media (Titan Biotech Ltd, Delhi, India). Total bacterial counts (Log colony forming unit (cfu)/g of paneer) were enumerated through incubating the plates anaerobically for 2 days at 37°C. The Y & M counts were done in the same manner (after 2 days at 30°C) using potato dextrose agar (Hi Media, Mumbai, India).

### Sensory evaluation of prepared paneer

Paneer was evaluated for its appearance and color, flavor, texture and overall acceptability with 9 points hedonic scale for each characteristic [21]. A panel of twenty people was selected including faculty members and students of Institute of Food Science and Nutrition.

### Total phenolic content

The total phenolic (TP) contents were determined by using Folin-Ciocalteu reagent method with some modifications [22]. One mL aliquot of WSEs of paneer was mixed with 1 mL of Folin-Ciocalteu reagent (10%) whereas for date extract and 1 mL whey was taken after dilution in the same manner. After vortexed the mixture, 2 mL of sodium carbonate (20%) solution was added. After incubation for 60 min at 30°C, the absorbance was measured at 760 nm using a spectrophotometer (Halo DB-20, UV-VIS double beam, Howard Way, Newport Pagnell, UK). Gallic acid (in ethanol) was used as a standard and the results of TPs were recalculated using gallic acid standard curve as μg gallic acid equivalent (GAE). All measurements were carried out in triplicates and the experiments were done in duplicate.

### Total flavonoids assay

Total flavonoids (TF) of each date extracts (~30°Brix) were determined according to Jia et al [23]. Aliquot (1.5 mL) of WSEs of paneer, date extract and whey was mixed with 75 μL of sodium nitrite (5%) solution. After vortexing (Scientific Industries Inc. Bohemia, NY, USA) for 1 min, 150 μL of aluminum chloride (10%) solution was added. Then after adding 0.5 mL of 1 mol/L NaOH, absorbance at 510 nm was measured using a spectrophotometer (Halo DB-20, UV-VIS double beam, UK). Catechin was used as a standard and the results were expressed using standard curve of catechin as μg catechin equivalent (CE). All determinations were carried out in triplicates and the experiments were done in duplicate.

### 2, 2-Diphenyl-1-picrylhydrazylradical scavenging activity assay

The capability of WSEs to scavenge 2, 2-diphenyl-1-picrylhydrazyl radical (DPPH^·^) was determined according to the method of Yiet al [24] with some modifications. Two mL of DPPH (60 μmol/L in absolute ethanol) solution was added into 2 mL of WSEs of paneer, diluted date extracts and whey. The mixture was vortexed (Scientific Industries Inc. USA) for 1 min. After incubation for 30 min at room temperature in dark, the absorbance was taken at 517 nm using a spectrophotometer (Halo DB-20, UV-VIS double beam, UK). The control (absolute ethanol) was also prepared in the same manner. The DPPH radical scavenging activity of date extract, paneer and whey was recalculated from the standard curve of Trolox as a μmol Trolox equivalent (TE). All determinations were carried out in triplicates and the experiments were done in duplicate.

### Trolox equivalent antioxidant capacity assay

For the determination of Trolox equivalent antioxidant capacity (TEAC), the WSEs were analyzed by using the method described by Prieto et al [25]. One mL of WSEs of paneer was mixed with 4 mL of reagent (0.6 mol/L sulphuric acid, 28 mmol/L sodium phosphate and 4 mmol/L ammonium molybdate) solution. Similarly, one mL of diluted date extracts and whey was run in the same manner. After incubating the mixture (95 min at 90°C), the absorbance was measured at 695 nm using a spectrophotometer. TEAC of date extract, paneer and whey was recalculated using Trolox standard curve as μmol TE. All determinations were carried out in triplicates and the experiments were done in duplicate.

### Reducing power or ferricyanide/Prussian blue assay

The reducing power (RP) of extracts of all WSEs was determined using the method as described by Reis et al [22] with some modifications. One mL of WSEs of paneer, diluted date extracts and whey was mixed with 0.5 mL each sodium phosphate buffer (0.2 mol/L, pH 6.6) and potassium ferricyanide (K_3_Fe(CN_6_)). After incubation the mixture (20 min, 50°C), 0.5 mL of trichloroacetic acid (%) was added and then vortexed (Scientific Industries Inc. USA). The mixture was centrifuged (Hermle Labortechnik GmbH Siemensstr-25 D-78564, Germany) at 3,000 g, 10 min, 4°C and clear supernatant was obtained. Then, 0.15 mL of ferric chloride (0.1%) was added to the supernatant. The absorbance of the mixture was measured using spectrophotometer (Halo DB-20, UV-VIS double beam, UK) at 700 nm. The RP ability was recalculated using standard curve of Trolox as μmol TE. The increased absorbance of the sample mixture depicted increased RP. All determinations were carried out in triplicates and the experiments were done in duplicate.

### Statistical analysis

Statistical analysis was performed through Minitab statistical software version 16 (Minitab Inc., State College, PA, USA), using the general linear model and Tukey’s test for pairwise comparison in analysis of variance at the level of p<0.05.

## RESULTS

### Physico-chemical characteristics of date cultivars

Physico-chemical characteristics of the investigated date cultivars are presented in Table 1. Rabi was observed to have maximum fruit weight (14.14 g) among the investigated date cultivars. Moisture, ash, crude fat, crude fiber, total protein, carbohydrate content, estimated energy values and acidity showed significant (p<0.05) variations between date cultivars. The highest pH value was shown by Rabi (6.00) whereas other cultivars showed values in the range 5.32 to 5.38. The highest moisture, ash and acidity were shown by Muzafati. The date cultivar having lowest content of moisture showed the highest estimated energy values. These energy values are attributed to the contents of carbohydrate. The more carbohydrates, the higher the estimated energy values and vice versa. Our results of moisture were in accordance with the results of date cultivars from Egypt and Oman, where moisture contents were reported in the range 17% to 30% [9,26]. The date cultivars from United Arab Emirates had carbohydrates in the range 67.33% to 75.30% whereas date cultivars from Oman had 73.66% to 82.44%. The range of calculated carbohydrates in the present study was 65.81% to 78.18% which was in agreement with the aforementioned results from Oman and United Arab Emirates. All the date cultivars contained very low content of protein, fat and fiber.

### Physico-chemical characteristics of milk and date extracts

Fresh milk was standardized for fat at 3.5%and SNF was calculated as 8.5±0.3. All the date extracts were maintained for ~30°Brix. Table 2 shows pH, acidity, vitamin C, reducing sugars and non-reducing sugars of date extracts. There was no significant (p<0.05) difference between the pH values of Rabi and Irani extracts. Aseel extract showed the lowest pH value (5.10). The acidity of all extracts ranged 0.41 to 0.54. Muzafati extract showed the maximum value (26.50 mg/100 mL) of vitamin C whereas the minimum value (13.80 mg/100 mL) was shown by Irani extract. Appreciable quantities of total sugars and reducing sugars were observed in Aseel and Irani extracts whereas the minimum values were observed in Rabi extract. Aseel extract showed the maximum values of non-reducing sugars.

### Antioxidant potential of date extracts

The extracts of Rabi, Irani and Aseel did not show significant (p<0.05) variations in their TP (μg GAE/mL of extract) and TF (μg CE/mL of extract) whereas Muzafati extract showed significantly (p<0.05) lower values compared to other cultivars extracts (Table 3). Nevertheless, Muzafati extract showed the minimum TP and TF but it showed the highest DPPH radical scavenging activity (1,180 μmol TE/mL of extract) which might be due to the presence of more potent antioxidant compounds. The minimum DPPH value was shown by Rabi extract. Aseel extract was found to have maximum TEAC (773.70 μmol TE/mL of extract) as compared to other cultivars extract but the extracts of Rabi, Muzafati and Irani did not show significant (p<0.05) variations in their TEAC. Although all the date extracts showed considerable values (μmol TE/mL of extract) of RP but there was no significant (p<0.05) variations in the values among different cultivars extracts.

### Proximal composition of prepared paneer

Table 4 shows the proximal composition of all paneer samples (treatments) prepared in the present study. The pH of all treatments significantly (p<0.05) decreased during storage. No significant (p<0.05) variations in pH values were observed between different treatments (paneer) until 4 days of storage but after 8 days, variations were significant. Our results were in accordance with the results of Das et al [27] who also observed the decreasing trend of pH of paneer during storage. The decrease in pH of paneer might be due to the activity of indigenous microorganisms which results in the accumulation of acids. The pH of whey after coagulation was 5.5±0.06 which was lower than the pH of all prepared paneer. Our results regarding moisture contents of all freshly prepared paneer were around 58% which were in accordance with the recommendations of Bureau of Indian Standards [28] i.e. to be below 60%. In a study conducted on Indian paneer, it was observed that it contained 56.25% moisture content which was near the values obtained in the present study [29]. Moisture contents of all paneer samples were significantly (p<0.05) varied between fresh samples and 8 days stored samples. A slight decreasing trend of moisture during storage was observed in all prepared paneer samples which might be due to loss of some moisture from the paneer matrix during storage. The decreasing trend of moisture during storage of paneer agrees with the results of Khatkar et al [30]. Fat contents of all the prepared paneer samples showed random trend during storage but there was no huge difference in the contents between all treatments. No significant (p<0.05) variations in the fat contents were found between fresh paneer from all treatments. Protein contents of all freshly prepared paneer samples did not vary significantly (p<0.05). Moreover, no significant difference in protein contents was observed during storage. Freshly prepared paneer samples of all treatments showed no significant (p<0.05) variations in their ash contents but significant variations were observed in values between fresh and 8 days stored samples. The ash contents of fresh paneer (~1.4%) prepared in the present study were in agreement with the results (1.4%) obtained by Hashmi et al [21]. No significant (p<0.05) variations were found in the values of fat on dry matter basis between all prepared paneer samples as well as during storage. Our results concerning fat on dry matter basis in paneer ranged 46.06 to 49.17 which were near the values recommended by Prevention of Food Adulteration Rules (PFA) [31].

### Microbiological quality of paneer

**Figure 1:**
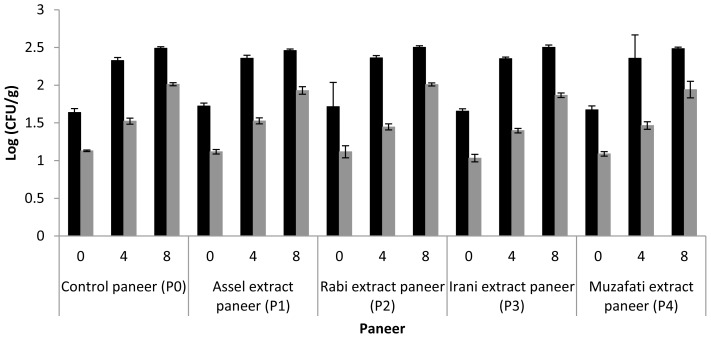
Changes in total plate counts (TPC) (black bars) and Yeast and Moulds (Y & M) (grey bars) of paneer during storage at 5°C.

Figure 1 shows the results of TPC and Y & M analysis. The microbiological quality of paneer may be dependent upon quality of milk, heat treatment of milk, unhygienic practices during manufacturing and its postmanufacture conditions like for instance, handling, packaging and storage. The results concerning TPC (Log cfu/g) of all treatments (paneer) were not significantly (p<0.05) different. During storage, the counts gradually increased in all treatments and no significant variations were found in TPC values of all treatments after 8 days of storage period. Similar trend was seen regarding Y & M counts. The values of Y & M also increased during storage in all treatments however, they were not significantly different from each other. It has been reported that usually coliforms, yeasts and moulds are completely destroyed during heating of milk at 82°C for 5 min but these organisms may be reinfected in the paneer during post manufacture conditions [32]. The increasing trend of TPC and Y & M counts during storage of paneer had also been reported in some studies [1,27]. The results of TPC and Y & M suggested that no inhibitory effect of date extracts which retain in the paneer matrix was shown against the microbes. So, microbes increased during storage in the treatments having date extracts. It was observed that fungus started to appear on the surface of paneer after 12 days of storage period. Our observations were concurrent with the observations of Das et al [27] who reported appearance of visible sliminess and reddish brown or yellowish brown discoloration on the surface of paneer after 12 days of storage. Such deteriorated paneer was discarded due to accumulation of microbes.

### Sensorial quality of prepared paneer

**Figure 2:**
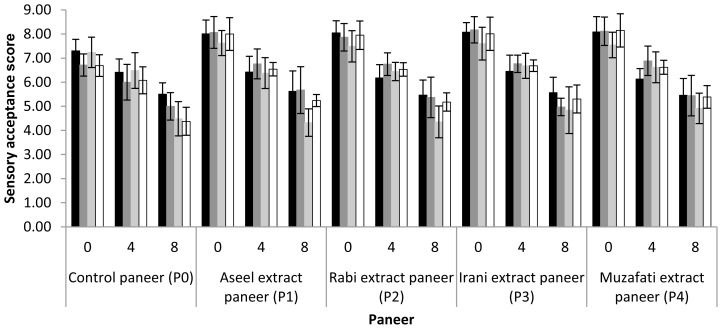
Sensory acceptance scores regarding appearance and color (black bars), flavor (dark grey bars), texture (light grey bars) and overall acceptability (white bars) of paneer during storage at 5°C.

Sensory attributes of any product determine its future. The suggested scores of different sensory attributes of paneer viz. appearance and color, flavor, texture and overall acceptability have been depicted in Figure 2. In general, all freshly prepared paneer supplemented with date extracts had higher scores of all sensory attributes compared to control treatment (paneer). Regarding appearance and color, flavor and overall acceptability, all the paneer supplemented with date extracts had higher score after 4 and 8 days of storage. The score of texture of paneer supplemented with Aseel, Rabi, and Muzafati date extracts did not vary significantly (p<0.05) from control paneer after 8 days of storage. The results of overall acceptability at all stages of storage period suggested that all paneer supplemented with date extracts were more acceptable compared to control treatment. But in general it was observed that the scores for all sensory attributes decreased during storage which was in agreement with results obtained by Singh et al [1] and Das et al [27]. Therefore, paneer stored for 12 days were not included in the sensory evaluation in the present study.

### Antioxidant potential of prepared paneer

The results concerning antioxidant potential of paneer prepared in the present study are shown in Table 5. Freshly prepared and 8 days stored paneer supplemented with different types of date extracts presented higher TP, TF, TEAC, and RP values than the control paneer (P_0_). The maximum values of TP (190.7 μg GAE/g paneer) were shown by the treatment P_2_. With the progress of storage period, TP, TF, DPPH radical scavenging activity, TEAC and RP values decreased in all prepared paneer. The maximum TF (479.4 μg CE/g of paneer) were present in freshly prepared P_3_ treatment. Owing to the presence of TF in date extracts, paneer supplemented with date extracts showed higher contents of TF than control paneer (P_0_). Even though, P_0_ showed TF but the contents (75.4 μg CE/g of paneer) were much less. Antioxidant activity of flavonoids has also been reported [33] but little is known regarding existence of flavonoids in dairy products. So presence of higher quantity of flavonoids in any dairy product or extracts may be due to the presence of some fruit or plant extracts rich in flavonoids.

Generally, DPPH radical scavenging activity of all prepared paneer was decreased during storage. Among freshly prepared paneer, P_0_ treatment showed minimum DPPH values (72.82 μmol TE/g of paneer) which might be due to presence of native milk proteins in paneer. In some studies, it has been reported that whey proteins in their hydrolysates were responsible for radical scavenging activity [34,35]. The maximum DPPH value (92.81 μmol TE/g of paneer) was shown by P_2_ treatment. By quickly reducing reactive oxygen species including free radicals, phenolic compounds are able to protect the biomolecules [36]. The hydroxy groups of flavonoids have the ability to donate hydrogen or electrons to DPPH free radicals which lead to termination reaction of those free radicals [37]. In this way, contributions of more hydroxy groups from phenolic and flavonoid compounds may increase the radical scavenging activity which was manifested in our results regarding date extract supplemented paneer.

The values (μmol TE/g of paneer) of TEAC obtained by all paneer supplemented with date extracts were higher than control paneer. Such high discrepancies of TEAC values might be due to presence of more flavonoids and phenolics in paneer supplemented with different date extracts. The freshly prepared control treatment showed very low value (25.10 μmol TE/g of paneer) of TEAC which also might be due to the presence of whey proteins in paneer [38]. The maximum values of TEAC were shown by P_2_ treatment (92.11 μmol TE/g of paneer) followed by P_3_ (84.12 μmol TE/g of paneer). The deterioration occurs in paneer with the increase in storage period and there might be the possibility of degradation of bioactive compounds in the paneer matrix during storage. Moreover, paneer does not contain starter or probiotic bacteria or some other useful microbes which are responsible for ripening, increase in bioactivity or inhibition of harmful microbes in the dairy products like cheese. Therefore, spoilage causing microbes find conducive environment to grow in and out of the paneer matrix. In addition, no preservative was used to increase the shelf life of paneer. Hence, it may be inferred that spoilage causing microbes cause degradation of bioactive compounds in the paneer matrix, leading to decrease in antioxidant potential during storage.

Similarly, RP of control paneer was very low compared to paneer supplemented with date extracts. The RP values (μmol TE/g of paneer) of freshly prepared paneer supplemented with date extracts were significantly decreased after storage period of 8 days. The maximum RP values (122.05 to 103.39 μmol TE/g of paneer) were shown by paneer supplemented with Aseel extract (P_1_) during storage. The assessment of RP of any extract may show its potential antioxidant activity. The reductive ability of any extract may indicate the presence of antioxidant compounds (phenolic and flavonoid compounds), responsible for the reduction of ferricyanide complex to ferrocyanide complex, which then reacts with ferric chloride to form ferric ferrous complex that shows maximum absorption at 700 nm in the assay [39]. On the basis of our results, it may be inferred that the paneer supplemented with date extracts may have potent antioxidant compounds which cause reduction of iron.

### Chemical characteristics of whey

Table 6 showed pH, acidity, vitamin C, reducing sugars and non-reducing sugars of whey obtained from all prepared paneer. The pH values observed in whey were lower than the values of paneer matrix. Higher acidity values of whey collected from paneer supplemented with date extracts were obtained compared to their respective extracts. The vitamin C contents of whey collected from control paneer were very low compared to the whey obtained from paneer supplemented with date extracts which might be due to entrance of considerable quantities of vitamin C into the whey from date extracts in all the treatments except control. Similarly, total sugars, reducing sugars and non-reducing sugars were also found in higher quantities in whey collected from paneer supplemented with date extracts compared to whey from control paneer.

### Antioxidant potential of whey

Table 7 presents the results regarding antioxidant potential of whey collected during manufacturing of different types of paneer. The TP and TF values of control paneer whey showed lower values than paneer supplemented with different types of date extracts. It means phenolics and flavonoids which were originally present in date extracts may enter into the whey of paneer from those date extracts. The values shown by control paneer whey concerning TP, TF, DPPH, TEAC, and RP were significantly different from whey collected from paneer supplemented with date extracts. The maximum values of TP, TF, and RP were shown by whey obtained from paneer supplemented with Irani extract. The TEAC values of whey obtained from all the paneer supplemented with date extracts were more than twice compared to the values of control paneer whey which might be due to presence of more antioxidant compounds (phenolics, flavonoids, and vitamin C) in the date extracts leaching into whey.

The whey from date extracts supplemented paneer was sweeter than whey from control paneer due to presence of enormous quantities of sugars. In this way, presence of vitamin C, total sugars in the whey from date extracts also offered a nutritious bye product of paneer. Whey is a byproduct of cheese containing bioactive components [35] but Pakistani people are unaware of its health benefits. It has been reported to be used in the preparation of functional milk beverage [35]. Therefore, keeping in view the health aspects of whey, it was also collected and analyzed for antioxidant potential in the present study. In short, it may be assumed that antioxidant potential of whey obtained from date extract supplemented paneer would be attributed to the presence of enormous quantities of whey proteins, some caseins and bioactive compounds originally present in date extracts. There is the possibility to use such whey as the basis of some nutritious food products.

## CONCLUSION

Among all the prepared paneer, paneer supplemented with Rabi extracts (P_2_) performed the best concerning TP, DPPH radical scavenging activity and TEAC. Generally, paneer supplemented with date extracts showed pronounced antioxidant activity due to presence of considerable quantities of phenolics and flavonoids. Such type of paneer also performed better regarding attributes related to their sensory characteristics.

On the basis of our findings, it may be suggested that the whey of such paneer, rich in potent antioxidants, may also be used in the formulation of functional foods.

## Figures and Tables

**Table 1:** Physico-chemical characteristics of date cultivars

Properties	Aseel	Rabi	Irani	Muzafati
Moisture (%)	22.09±0.58^b^	18.45±0.31^c^	17.22±0.34^d^	28.17±0.58^a^
Fruit weight (g)	9.62±1.28^b^	14.14±1.54^a^	7.13±0.59^c^	6.88±1.50^c^
Ash (%)	1.67±0.06^d^	2.19±0.09^b^	1.92±0.05^c^	2.29±0.06^a^
Protein (%)	0.70±0.06^d^	0.98±0.04^c^	1.28±0.05^a^	1.15±0.05^b^
Fat (%)	1.31±0.05^a^	1.18±0.05^b^	1.11±0.06^c^	0.62±0.04^d^
Fiber (%)	0.49±0.05^c^	1.11±0.02^b^	0.29±0.04^d^	1.96±0.10^a^
Carbohydrates (%)	73.74±0.60^c^	76.09±0.32^b^	78.18±0.31^a^	65.81±0.62^d^
Energy (kcal/100 g)	309.57±2.42^c^	318.90±1.34^b^	327.79±1.41^a^	273.41±2.40^d^
pH	5.35±0.04^b^	6.00±0.03^a^	5.32±0.03^b^	5.38±0.05^b^
Acidity (%)	1.27±0.06^b^	1.02±0.07^c^	0.76±0.06^d^	1.45±0.07^a^

a–d Means with different letters in the same column show significant (p<0.05) differences between treatments.

**Table 2:** pH, acidity, vitamin C, total sugars, reducing sugars and non-reducing sugars of date extracts

Date cultivars	pH	Acidity (%)	Vitamin C1)	Total sugars (%)	Reducing sugars (%)	Non-reducing sugars (%)
Aseel extract	5.10±0.04^c^	0.54±0.03^a^	21.40±1.25^b^	26.40±1.08^a^	21.30±0.68^a^	5.20±1.36^a^
Rabi extract	6.00±0.03^a^	0.41±0.02^c^	19.70±0.75^b^	16.00±0.75^c^	13.00±0.32^b^	3.00±0.77^b^
Irani extract	5.20±0.03^b^	0.52±0.03^ab^	13.80±0.85^c^	23.50±1.07^b^	21.70±0.95^a^	1.80±0.18^b^
Muzafati extract	5.90±0.04^a^	0.47±0.02^bc^	26.50±0.95^a^	17.60±1.07^c^	14.40±0.85^b^	3.10±0.30^ab^

1) mg/100 g of extract.

a–c Means with different letters in the same column show significant (p<0.05) differences between treatments.

**Table 3:** Total phenolics, total flavonoids, radical scavenging activity, Trolox equivalent antioxidant capacity, and reducing power of date extracts

Date cultivars	TP	TF	DPPH	TEAC	RP
Aseel extract	3,340.00±131.20^a^	9,060.00±789.00^a^	1,120.00±220^ab^	773.70±33.10^a^	1,834.30±62.50^a^
Rabi extract	3,600.00±137.50^a^	8,777.00±713.00^a^	720.00±30^b^	675.30±22.50^b^	1,856.00±63.60^a^
Irani extract	3,320.00±131.10^a^	9,040.00±913.00^a^	1,090.00±210^ab^	668.30±28.40^b^	1,787.00±84.60^a^
Muzafati extract	2,740.00±88.90^b^	4,770.00±430.00^b^	1,180.00±170^a^	621.00±37.60^b^	1,726.70±62.50^ab^

TP, total phenolics (μg gallic acid equivalent/mL extract); TF, total flavonoids (μg catechin equivalent/mL extract); DPPH, 2, 2-diphenyl-1-picrylhydrazyl radical (DPPH) (μmol equivalent of Trolox/mL extract); TEAC, Trolox equivalent antioxidant capacity (μmol equivalent of Trolox/mL extract); RP, reducing power (μmol equivalent of Trolox/mL extract).

a,b Means with different letters in the same column show significant (p<0.05) differences between date extracts.

**Table 4:** Proximal characteristics of paneer supplemented with different date extracts during storage

Treatments	Days	pH	Moisture (%)	Fat (%)	Protein (%)	Ash (%)	Fat/dry matter (%)
Control paneer (P_0_)	0	5.94±0.04^a^	58.91±0.51^a^	19.03±0.23^e^	18.63±0.27a	1.43±0.30^a^	46.32±0.17^a^
	4	5.61±0.06^b^	57.91±0.51abcde	19.53±0.25^de^	19.31±0.22a	1.24±0.05cde	46.41±0.07^a^
	8	5.41±0.02^cd^	56.91±0.51def	20.33±0.25bcd	19.59±0.67a	1.15±0.02^e^	47.20±1.02^a^
Aseel extract paneer (P_1_)	0	5.91±0.02^a^	58.54±0.61^ab^	20.05±0.72bcde	18.17±0.96a	1.43±0.01^a^	48.39±2.34^a^
	4	5.64±0.04^b^	57.75±0.25abcde	20.33±0.20bcd	18.63±0.39a	1.27±0.04bcd	48.13±0.72^a^
	8	5.25±0.05^de^	56.75±0.25^ef^	20.76±0.15abc	19.32±0.39a	1.15±0.01^e^	48.02±0.63^a^
Rabi extract paneer (P_2_)	0	5.92±0.03^a^	58.34±0.19abc	19.86±0.20cde	18.76±0.35a	1.40±0.01^a^	47.69±0.71^a^
	4	5.56±0.02^bc^	57.34±0.19bcdef	20.50±0.10abcd	18.77±0.33a	1.38±0.06^ab^	48.06±0.41^a^
	8	4.75±0.04^f^	56.34±0.19^f^	21.47±0.15^a^	18.77±0.09a	1.41±0.03^a^	49.17±0.16^a^
Irani extract paneer (P_3_)	0	5.84±0.08^a^	58.24±0.19abcd	19.83±0.35cde	18.57±0.30a	1.35±0.05abc	47.50±0.76^a^
	4	5.57±0.08^bc^	57.98±0.88abcde	20.36±0.15abcd	18.31±0.72a	1.33±0.06abc	48.48±0.68^a^
	8	4.79±0.08^f^	56.98±0.88cdef	21.03±0.15^ab^	18.84±0.86^a^	1.14±0.02^e^	48.91±0.99^a^
Muzafati extract paneer (P_4_)	0	5.89±0.08^a^	58.67±0.16^ab^	19.03±0.70^e^	18.86±0.81^a^	1.42±0.01^a^	46.06±1.80^a^
	4	5.56±0.13^bc^	58.04±0.19abcde	19.66±0.60cde	19.04±0.57^a^	1.24±0.03cde	46.87±1.41^a^
	8	5.14±0.04^e^	57.04±0.19cdef	20.30±0.40bcd	19.48±0.38^a^	1.17±0.01^de^	47.25±0.90^a^

a–f Means with different letters in the same column show significant (p<0.05) differences between treatments and storage period.

**Table 5:** The changes in total phenolics, total flavonoids, radical scavenging activity, trolox equivalent antioxidant capacity, and reducing power of paneer supplemented with different date extracts during storage

Treatments	Days	TP	TF	DPPH	TEAC	RP
Control paneer (P_0_)	0	119.2±16.29^de^	75.4±9.32^g^	72.82±1.56^c^	25.10±0.6^i^	16.21±0.56^g^
	4	56.3±6.11^gh^	23.3±5.92^h^	45.26±2.15^ef^	21.03±0.7^j^	14.95±0.66^g^
	8	24.9±3.49^h^	24.6±3.86^h^	15.55±0.79^j^	13.01±0.3^k^	14.29±0.91^g^
Aseel extract paneer (P_1_)	0	172.6±9.36abc	410.6±8.66^b^	78.43±1.79^b^	79.04±0.8^d^	122.05±6.05^a^
	4	112.7±9.12^de^	176.7±5.28^d^	49.06±0.81^d^	76.05±1.0^e^	113.17±2.17^b^
	8	107.3±15.86^ef^	62.7±4.57^g^	37.72±0.83^g^	54.10±1.3^f^	103.39±3.08^c^
Rabi extract paneer (P_2_)	0	190.7±11.27^a^	158.3±6.44^e^	92.81±2.64^a^	92.11±1.5^a^	116.57±5.08^ab^
	4	145.6±10.54bcd	141.8±4.17^e^	47.55±1.28^de^	87.11±2.0^b^	112.10±2.29^b^
	8	114.3±7.41^de^	77.0±4.49^g^	43.74±0.93^ef^	39.01±0.8^h^	85.23±2.81^e^
Irani extract paneer (P_3_)	0	176.7±9.25^ab^	479.4±5.43^a^	76.86±0.55^bc^	84.12±1.4^bc^	114.59±3.24^ab^
	4	141.3±12.43^cd^	197.4±5.58^c^	41.21±1.09^fg^	83.10±0.7^c^	92.28±1.99^e^
	8	102.2±20.10^ef^	110.6±6.16^f^	21.99±1.55^i^	46.08±1.1^g^	86.20±1.45^e^
Muzafati extract paneer (P_4_)	0	115.2±10.35^de^	208.1±7.26^c^	74.58±1.25^bc^	77.03±1.2^de^	102.22±2.34^c^
	4	97.0±5.34^ef^	101.7±4.78^f^	37.82±0.64^g^	52.02±1.3^f^	100.56±2.75^cd^
	8	75.3±6.94^fg^	65.8±2.77^g^	31.24±1.30^h^	36.20±1.0^h^	42.80±1.55^f^

TP, total phenolics (μg gallic acid equivalent/g paneer), TF, total flavonoids (μg catechin equivalent/g of paneer); DPPH, 2, 2-diphenyl-1-picrylhydrazyl radical (μmol equivalent of Trolox/g of paneer); TEAC, Trolox equivalent antioxidant capacity (μmol equivalent of Trolox/g of paneer); RP, reducing power (μmol equivalent of Trolox/g of paneer).

a–k Means with different letters in the same column show significant (p<0.05) differences between treatments and storage period.

**Table 6:** pH, acidity, vitamin C, total sugars, reducing sugars and non-reducing sugars of whey from paneer supplemented with different date extracts

Date cultivars	pH	Acidity (%)	Vitamin C1)	Total sugars (%)	Reducing sugars (%)	Non-reducing sugars (%)
Control paneer whey	5.23±0.02^a^	0.43±0.06^d^	1.33±0.15^e^	3.98±0.46^c^	2.26±0.14^d^	1.72±0.38^c^
Aseel extract paneer whey	5.14±0.01^c^	0.57±0.03^c^	19.26±0.72^b^	13.93±0.56^a^	11.81±0.35^A^^a^	2.11±0.36^bc^
Rabi extract paneer whey	5.19±0.01^b^	0.97±0.01^a^	16.25±0.75^c^	13.31±0.24^a^	10.11±0.43^b^	3.20±0.20^a^
Irani extract paneer whey	5.16±0.02^bc^	0.53±0.06^cd^	9.78±0.12^d^	11.20±0.56^b^	8.28±0.16^c^	2.92±0.68^ab^
Muzafati extract paneer whey	5.23±0.01^a^	0.85±0.03^b^	21.59±1.36^a^	13.97±0.57^a^	10.12±0.55^b^	3.85±0.10^a^

1) mg/100 g of extract.

a–e Means with different letters in the same column show significant (p<0.05) differences between treatments.

**Table 7:** Total phenolics, total flavonoids, radical scavenging activity, trolox equivalent antioxidant capacity, and reducing power of whey from paneer supplemented with different date extracts

Treatments	TP	TF	DPPH	TEAC	RP
Control paneer whey	0.34±0.01^c^	1.24±0.05^d^	06.40±0.03^d^	5.12±0.09^c^	2.59±0.09^d^
Aseel extract paneer whey	1.21±0.05^ab^	7.11±0.09^b^	12.10±0.03^a^	12.69±0.02^b^	4.59±0.06^b^
Rabi extract paneer whey	1.31±0.03^a^	6.59±0.08^c^	10.50±0.08^b^	13.71±0.05^a^	4.34±0.09^c^
Irani extract paneer whey	1.33±0.07^a^	7.62±0.11^a^	08.70±0.03^c^	13.44±0.09^a^	4.90±0.06^a^
Muzafati extract paneer whey	1.10±0.06^b^	7.32±0.09^b^	10.80±0.07^ab^	12.62±0.06^b^	4.32±0.05^b^

TP, total phenolics (μg gallic acid equivalent/mL whey); TF, total flavonoids (μg catechin equivalent/mL whey); DPPH, 2, 2-diphenyl-1-picrylhydrazyl radical (μmol equivalent of Trolox/mL whey); TEAC, Trolox equivalent antioxidant capacity (μmol equivalent of Trolox/mL whey); RP, reducing power (μmol equivalent of Trolox/mL whey).

a–d Means with different letters in the same column show significant (p<0.05) differences between treatments.
